# Primary Chondroblastic Osteosarcoma of the Lung

**DOI:** 10.1080/13577149977640

**Published:** 1999-12

**Authors:** Monica S. Wagner, Cesar V. Reyes

**Affiliations:** 1 Pathology & Laboratory Service Veterans Affairs Hospital Hines Illinois USA; 2 Department of Pathology Loyola University Medical Center Maywood Illinois USA; 3 Room D121 P.O. Box 5000 Fifth Avenue & Roosevelt Road Hines IL 60141 USA

## Abstract

*Purpose.* Primary extra-osseous osteosarcomas are uncommon lesions, and those originating within the lung are especially rare, with few case reports existing in the literature.

*Patient.* We report the case of a 48-year-old male smoker with a primary osteosarcoma of the right lower lung lobe.

*Results.* Diagnosis was based on histopathological findings of a poorly differentiated sarcoma with malignant cellular components of osteoid and chondroid matrix, along with immunohistochemical and electron microscopy confirmation. Extensive clinical and radiographic evaluation failed to reveal any tumor at other anatomical sites.


*Sarcoma* (1999) **3**, 193–195

CASE REPORT

### Primary chondroblastic osteosarcoma of the lung

MONICA S. WAGNER & CESAR V. REYES

*Pathology & Laboratory Service, Veterans Affairs Hospital, Hines, and Department of Pathology, Loyola University Medical Center, Maywood, Illinois, USA*

**Abstract**

*Purpose.* Primary extra-osseous osteosarcomas are uncommon lesions, and those originating within the lung are especially rare, with few case reports existing in the literature.

*Patient.* We report the case of a 48-year-old male smoker with a primary osteosarcoma of the right lower lung lobe.

*Results.* Diagnosis was based on histopathological findings of a poorly differentiated sarcoma with malignant cellular components of osteoid and chondroid matrix, along with immunohistochemical and electron microscopy confirmation. Extensive clinical and radiographic evaluation failed to reveal any tumor at other anatomical sites.

### Patient

A 48-year old male presented with chest pain and a right pleural effusion. A computer tomographic scan demonstrated a cystic calcified lung mass of the right lower lobe (Fig. 1). His past medical history was significant for insulin-dependent diabetes mellitus, end-stage renal disease, and multiple cerebrovascular accidents. The patient served in Vietnam combat, smoked a pack of cigarettes per day throughout his adult lifetime, and worked in the carpentry field.

A bronchial washing cytology and fine-needle aspiration (FNA) cytology of the right lung lesion

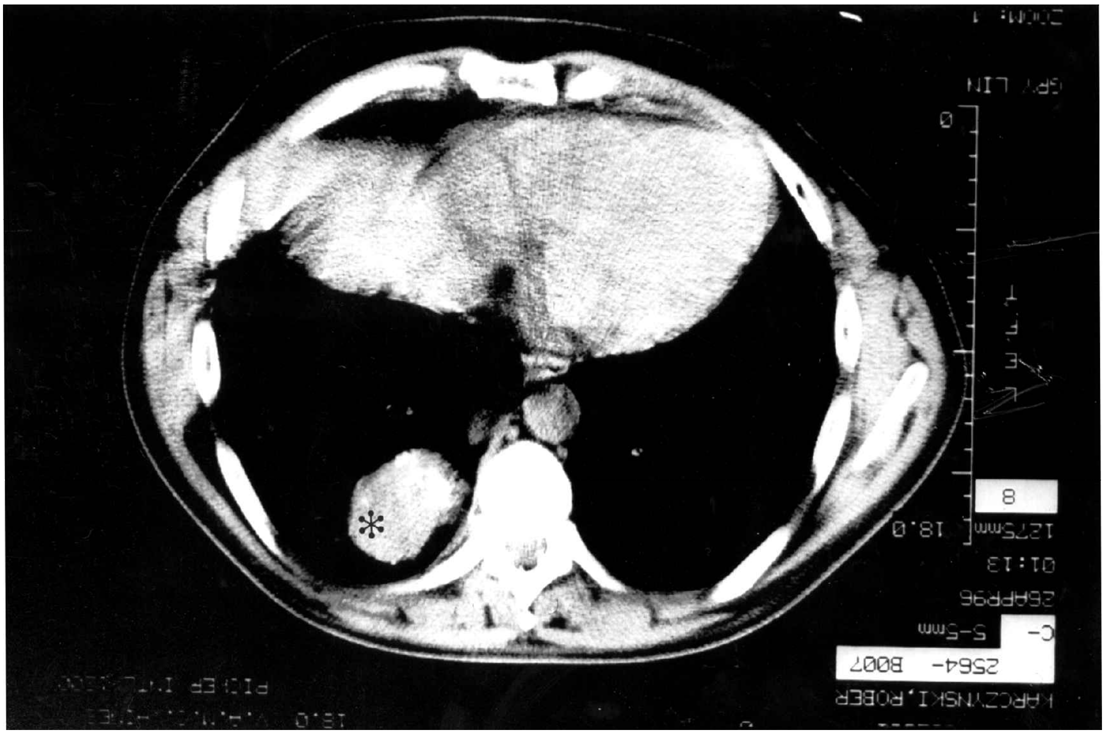

**Figure 1**. *The computer tomographic scan reveals a calcified, extra-osseous tumor mass (*) in the lung.*

Correspondence to: C.V. Reyes, 78–113, Room D121, P.O. Box 5000, Fifth Avenue & Roosevelt Road, Hines, IL 60141, USA. Tel. (708) 202-VETS x21295; Fax (708) 216-2588; Email acvrear@aol.com

1357-714X print/1369-1643 online/99/040193-03 © 1999 Taylor & Francis Ltd showed necrosis and rare isolated malignant cells. Subsequent tissue biopsy of the right lung and pleura reaffirmed that the tumor was a highly cellular and poorly differentiated sarcoma (Fig. 2), composed of spindle and epithelioid cells with a brisk rate of mitoses, occasional fascicular streaming pattern, few multinucleated giant cells consistent with osteoclasts, and frequent areas of necrosis. Pale islands of malignant cells forming chondroid, as well as more eosinophilic areas of malignant osteoid matrix (Fig. 3) with immature bone were present throughout a background of fibrosarcoma-like stroma. Approximately 25% of the tumor showed areas of malignant cartilage.

Special studies and immunohistochemical staining revealed strongly positive vimentin, weakly positive alpha-fetoprotein (AFP), focally positive lysozyme and *beta*-human chorionic gonadotropin (*b*HCG), negative epithelial markers, including various keratins and epithelial membrane antigen (EMA), negative desmin, and negative carcino-embryonic antigen (CEA). Although there was immunohistochemical detection of AFP and bHCG, a diagnosis of malignant teratoma was ruled out due to absence of embryonic and other tissue elements.

Electron microscopy studies demonstrated malignant fibroblastic cells with abundant intercellular matrix consistent with osteoid stroma. Based on cellular, immunohistochemical, and ultrastructural findings, the diagnosis of chondroblastic osteosarcoma was made.

Subsequent extensive clinical work-up by multiple radiographic imaging studies and complete physical examinations failed to reveal any further focus of the tumor. Therefore, all findings supported an osteosarcoma originating from within the lung itself. The patient died 1 month after diagnosis; autopsy was not authorized.

### Discussion

Osteosarcomas are highly malignant lesions, uncommonly originating from extra-osseous sites. The lung is a particularly rare site of origin for this type of tumor.

The first instance of a primary 'osteoid chondrosarcoma' of the lung was reported by Greenspan in 1933.[1] Greenspan identified this lesion at autopsy in a 35-year-old woman with no evidence of further malignancy, so concluded that this lesion could arise from an intrapulmonary site. To the best of our knowledge, since that time only nine additional cases of confirmed primary osteosarcomas of the lung are noted within the literature.[2–10]

In definitively identifying a lung lesion as a primary osteosarcoma, several criteria must be met. Perhaps the largest problem is incorrectly categorizing tumors as osteosarcomas, rather than carcinosarcomas. Negative EMA and cytokeratin staining and electron microscopic absence of desmosomes and intercellular junction structures help to confirm that there is no epithelial differentiation and that the lesion is truly a sarcoma. One study examining pulmonary osteosarcomas by immunohistological studies, for example,

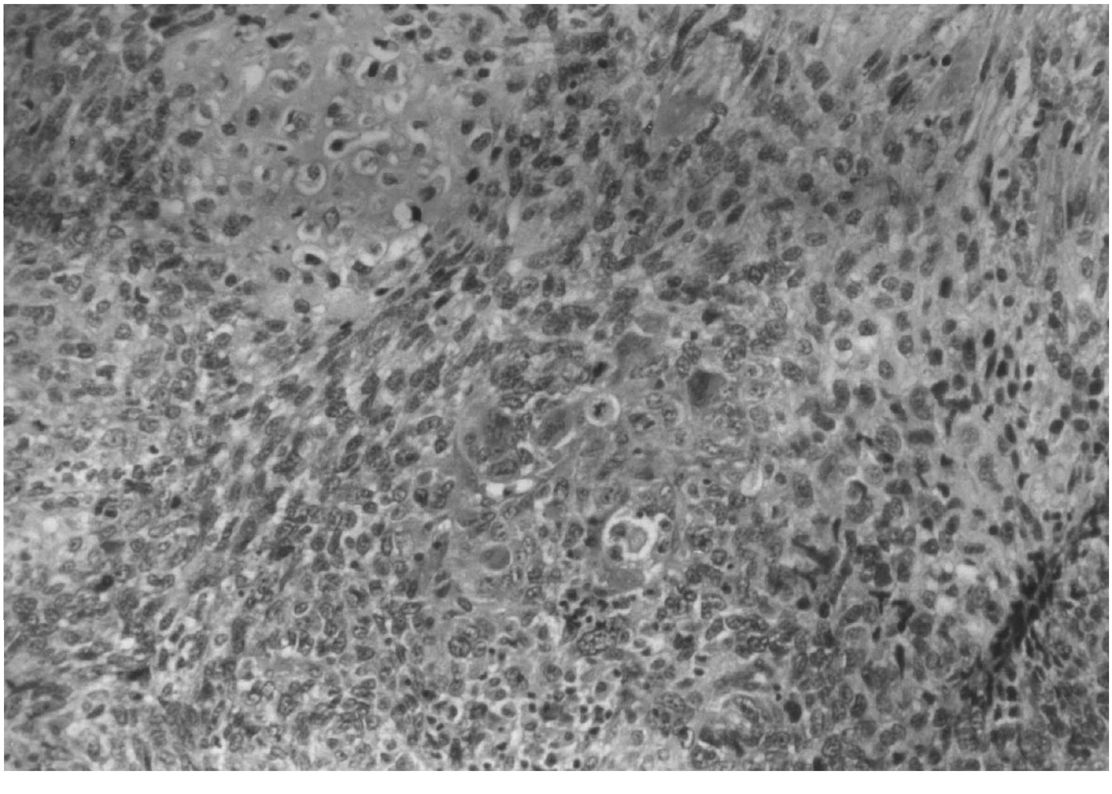

**Figure 2.** *Microscopically, the neoplasm is highly cellular, sarcomatous, pleomorphic with chondroid and osteoclastic components. Hematoxylin eosin stain, ×100.*

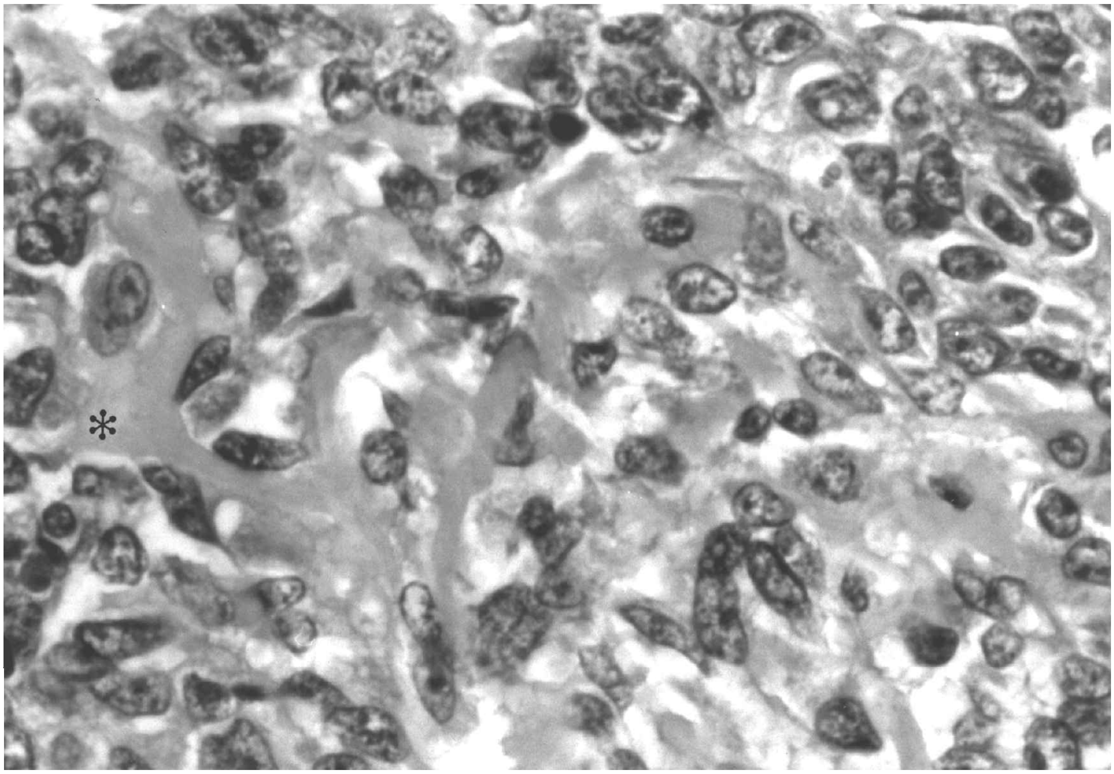

**Figure 3.** *Osteoid matrix (*) is also prominent. Hematoxylin eosin stain, ×200.*

led to the reclassification of two out of five cases as carcinosarcomas, based on identification of epithelial elements with positive staining by EMA and cytokeratin.[2]

Besides being a rare pulmonary lesion in itself, this case of osteosarcoma is unique in the extensive amount of malignant chondroblastic tissue it contains within its parenchyma. In comparison to the previously identified cases, it fits the standard of being a single mass in an adult patient with no extrapulmonary manifestations of the disease. On extensive tissue sampling and special studies, no foci of carcinomatous differentiation were identified.

Despite improvements in multimodality therapy in recent years, the prognosis for extra-osseous osteogenic sarcomas remains poor. Nevertheless, it is important to consider these rare tumors in the differential diagnosis of extra-osseous tumors demonstrating osteoid features.

### References

1 Greenspan EB. Primary osteoid chondrosarcoma of the lung. Report of a case. *Am J Cancer* 1933;18:603–9.

2 Colby TV, Bilbao JE, Battifora H, Unni KK. Primary osteosarcoma of the lung. A reappraisal following immunohistologic study. *Arch Pathol Lab Med* 1989;113:1147–50.

3 Yamashita T, Kiyota T, Ukishima G, Hayama T, Yabuki E. Autopsy case of chondro-osteoidsarcoma originating in the lung. *J Showa Med Assoc* 1964;22:472–3.1.

4 Nosanchuk JS, Weatherbee L. Primary osteogenic sarcoma in the lung: report of a case. *J Thorac Cardiovasc Surg* 1969;58:242–7.

5 Reingold IM, Amromin GD. Extraosseous osteosarcoma of the lung. *Cancer* 1971;28:491–8.2.

6 Nascimento AG, Unni KK, Bernatz PE. Sarcomas of the lung. *Mayo Clin Proc* 1982;57:355–9.

7 Petersen, M. Radionucleotide detection of primary pulmonary osteogenic sarcoma: a case report and review of the literature. *J Nuc Med* 1990;131:1110–14.1.

8 Bhalla M, Thompson BG, Harley RA, McLoud TC. Primary extraosseous pulmonary osteogenic sarcoma: CT findings. *J Comp Assist Tomography* 1992;16:974–6.

9 Loose JH, El-Naggar AK, Ro JY, Huang W, McMurtrey MJ, Ayala AG. Primary osteosarcoma of the lung. *J Thorac Cardiovasc Surg* 1990;100:867–73.

10 Kalengayi R, De Francquen P, Vanderhoeft P, Parmentier R. Un cas de'osteosarcome primitif du poumon. *Ann Anat Pathol* 1970;15:221–30.

## References

[PDF_00137] Bhalla M., Thompson B. G., Harley R. A., McLoud T. C. (1992). Primary extraosseous pulmonary osteogenic sarcoma: CT findings.. J Comput Assist Tomogr.

[PDF_00120] Colby T. V., Bilbao J. E., Battifora H., Unni K. K. (1989). Primary osteosarcoma of the lung. A reappraisal following immunohistologic study.. Arch Pathol Lab Med.

[PDF_00145] Kalengayi R., de Francquen P., Vanderhoeft P., Parmentier R. (1970). Un cas d'ostéosarcome primitif du poumon.. Ann Anat Pathol (Paris).

[PDF_00141] Loose J. H., el-Naggar A. K., Ro J. Y., Huang W. L., McMurtrey M. J., Ayala A. G. (1990). Primary osteosarcoma of the lung. Report of two cases and review of the literature.. J Thorac Cardiovasc Surg.

[PDF_00132] Nascimento A. G., Unni K. K., Bernatz P. E. (1982). Sarcomas of the lung.. Mayo Clin Proc.

[PDF_00127] Nosanchuk J. S., Weatherbee L. (1969). Primary osteogenic sarcoma in lung. Report of a case.. J Thorac Cardiovasc Surg.

[PDF_00134] Petersen M. (1990). Radionuclide detection of primary pulmonary osteogenic sarcoma: a case report and review of the literature.. J Nucl Med.

[PDF_00130] Reingold I. M., Amromin G. D. (1971). Extraosseous osteosarcoma of the lung.. Cancer.

[PDF_00124] YAMASHITA T., KIYOTA T., UKISHIMA G., HAYAMA T., YABUKI E. (1964). [AUTOPSY CASE OF CHONDRO-OSTEOIDSARCOMA ORIGINATING IN THE LUNG].. Showa Igakkai Zasshi.

